# Evidence from internet search data shows information-seeking responses to news of local COVID-19 cases

**DOI:** 10.1073/pnas.2005335117

**Published:** 2020-05-04

**Authors:** Ana I. Bento, Thuy Nguyen, Coady Wing, Felipe Lozano-Rojas, Yong-Yeol Ahn, Kosali Simon

**Affiliations:** ^a^School of Public Health, Indiana University, Bloomington, IN 47405;; ^b^O’Neill School of Public and Environmental Affairs, Indiana University, Bloomington, IN 47405;; ^c^Luddy School of Informatics, Computing and Engineering, Indiana University, Bloomington, IN 47408

**Keywords:** COVID-19, Google Trends, information

## Abstract

The COVID-19 outbreak is a global pandemic with community circulation in many countries, including the United States, with confirmed cases in all states. The course of this pandemic will be shaped by how governments enact timely policies and disseminate information and by how the public reacts to policies and information. Here, we examine information-seeking responses to the first COVID-19 case public announcement in a state. Using an event study framework for all US states, we show that such news increases collective attention to the crisis right away. However, the elevated level of attention is short-lived, even though the initial announcements are followed by increasingly strong policy measures. Specifically, searches for “coronavirus” increased by about 36% (95% CI: 27 to 44%) on the day immediately after the first case announcement but decreased back to the baseline level in less than a week or two. We find that people respond to the first report of COVID-19 in their state by immediately seeking information about COVID-19, as measured by searches for coronavirus, coronavirus symptoms, and hand sanitizer. On the other hand, searches for information regarding community-level policies (e.g., quarantine, school closures, testing) or personal health strategies (e.g., masks, grocery delivery, over-the-counter medications) do not appear to be immediately triggered by first reports. These results are representative of the study period being relatively early in the epidemic, and more-elaborate policy responses were not yet part of the public discourse. Further analysis should track evolving patterns of responses to subsequent flows of public information.

The first confirmed case of novel coronavirus disease 2019 (COVID-19) in the United States occurred in Washington State on January 21, 2020. Since then, the virus has spread across the country (1, 2). There are confirmed cases in every state, and the vast majority are not connected to international travel (3), indicating that the virus had been circulating for several weeks before the first positive test (4, 5). Slowing the transmission of the virus will help reduce the burden of the disease, save lives, and reduce strain on the health care system (4, 6). In the absence of a vaccine, the main strategies for reducing transmission involve sanitation and handwashing, disciplined social distancing, quarantines, and school and workplace closures (6). These nonpharmaceutical interventions (NPIs) are effective and powerful ways to control transmission (7), as shown by earlier epidemics (8) and the steady decline of COVID-19 cases in China (2). To be successful, NPIs require people to undertake behavioral changes that may be personally costly. This is particularly true in countries with deeply rooted norms about personal freedom and reluctance to impose mandatory policies.

In settings where restrictions on the free movement of residents are less strictly enforced, it is especially critical for public officials to know whether and for how long statements from leaders motivate individuals to efficiently seek and absorb information, as officials put their communication strategies in place. Such coordinated actions are needed at the first signs of community spreading and cannot be guided through traditional polling methods, which take too long. Because the extent of the epidemic is likely to be underestimated at this stage, it is crucial to know how much the early case announcements induce heightened collective attention and information-seeking behavior.

To provide rapid evidence to inform policy making, we use internet search data in an event study design to examine how collective attention and information-seeking behaviors respond to state government announcements of first COVID-19 cases. We highlight changes in search patterns occurring in the days leading up to and following first case announcements in a state (1, 2).

## Results

**Fig. 1.**
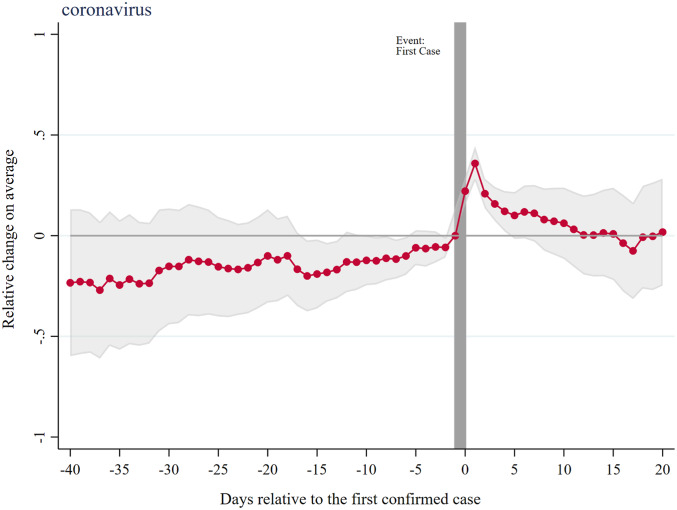
Time-varying effects of announcements of the first COVID-19 case in a state on searches for coronavirus. The period prior to the treatment (first confirmed case) is set as a reference: gray vertical bar. In red are the estimated coefficients (95% CI, gray band) in the Poisson model (differences in log-expected counts of search relative to the period prior to the event). The average search frequency of this term is 97,023.9 per state per day.

Fig. 1 depicts the event study estimates of the effects of initial announcements on overall coronavirus search. Searches for “coronavirus” increased by about 36% (95% CI: 27 to 44%) on the day immediately after the announcement but quickly decreased back to the baseline level in less than a week or two (Fig. 1). There was no observable trend in the search behavior in the days leading up to the announcements, suggesting the first “local” case indeed heightened the collective attention to the pandemic. However, the increased level of information seeking faded within 2 wk, even though many announcements of school closures or other mitigation strategies followed, suggesting that increased attention is only short-lived. This is consistent with no observable trend in the sense of urgency prior to the local announcement.

**Fig. 2.**
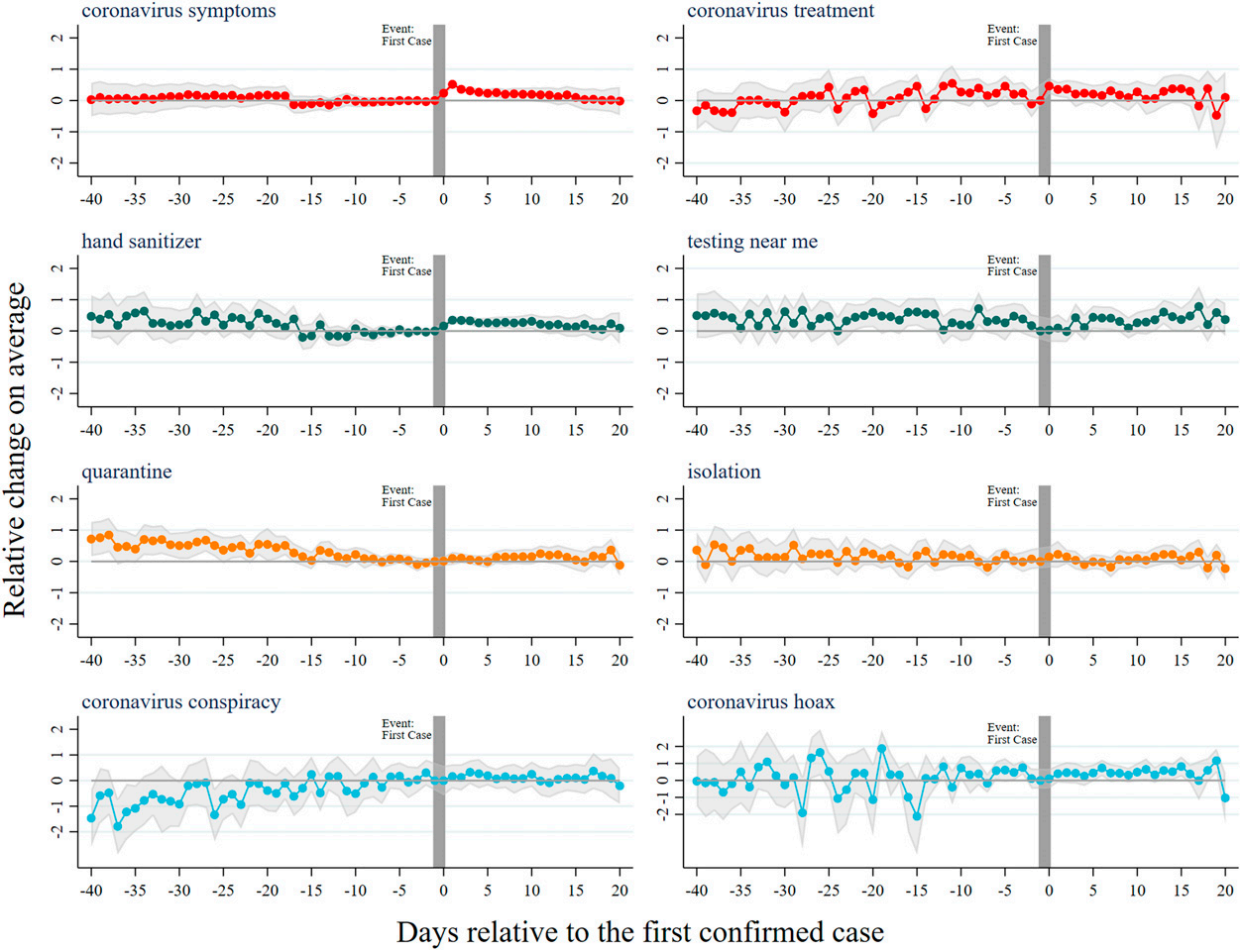
Time-varying effects of announcements of the first COVID-19 case in a state on searches for 1) symptoms and treatments (red), 2) hand sanitizer and diagnostic tests (green), 3) coordinated responses (orange), and 4) narratives that undermine public responses (blue). Point estimated coefficients are the dotted lines (95% CI, gray band) in the Poisson models (differences in log-expected counts of search relative to the period prior to the event). Average search frequency per state per day for the terms: coronavirus symptoms (8,522.6), coronavirus treatment (448.2), hand sanitizer (3,214.7), testing near me (381.6), quarantine (2,158.2), isolation (631.2), coronavirus conspiracy (205.1), and coronavirus hoax (123.0).

Fig. 2 shows the event studies of internet search for 1) symptoms and treatments, 2) hand sanitizer and diagnostic tests, 3) coordinated responses, and 4) narratives that undermine public responses. News of the first COVID-19 case in a state leads to a 52% increase in searches for “coronavirus symptoms” but does not increase searches for coronavirus treatment options. The second row shows searches for “hand sanitizer” increasing 35% immediately after first case announcements, and, unlike in the previous two cases, the search activity remains high for the remainder of the observable period. However, the announcements do not induce searches for nearby coronavirus testing opportunities, at least in the period under study. The third row suggests that first case announcements do not induce search for community-level policies (quarantines, school closures, and coronavirus testing) or more-elaborate personal health strategies (face masks, grocery delivery, over-the-counter medications). The final row examines how first case reports affect searches about the credibility of the epidemic. There is no indication that government confirmation of the first case increases or reduces searches for coronavirus hoaxes and overreactions; one might have expected official news to reduce concerns about false news.

### Robustness Checks.

To investigate information spillovers in which states respond to epidemics in other states, we examined augmented models that controlled for the timing of the announcements of Washington, Illinois, California, and Arizona, as well as for the timing of the earliest announcement among a state’s neighbor states [section A of additional results at https://github.com/anabento/GoogleBehaviorCovid (9)]. The results show that COVID-19 searches responded to Washington’s first case (Dataset S1, column 2), to first case announcements in other states that occurred in January (Dataset S1, column 3), and to announcements in neighboring states (Dataset S1, column 4). These results indicate some spillovers, but the main conclusions regarding the effect of a state’s own first case remains. Each state may experience different treatment effects of the first COVID-19 cases on online searches. Therefore, we present single-state time series of search volumes in figure B-2 in additional results available at https://github.com/anabento/GoogleBehaviorCovid (9), to descriptively depict the treatment effect heterogeneity. The results suggest that information-seeking responses were larger and faster in late states compared to the early states. We checked whether the findings were driven by accessed date of the data by rerunning the analysis using the latest search output from Google Trends, which we retrieved on April 18, 2020 and compared to the current results using the data retrieved on March 20, 2020 [https://github.com/anabento/GoogleBehaviorCovid (9)]. These core results were not sensitive to this replication.

## Conclusion

Our results suggest that first state COVID-19 case announcements do lead to a widespread increase in the extent to which people seek out information about the epidemic. We find that announcements of first cases have the biggest effect on searches for basic information about the virus and its symptoms, suggesting that people attempt to educate themselves about COVID-19 and its effects. However, first case reports do not lead to a differential increase in searches involving large-scale NPIs (e.g., school and workplace closures) that will likely have important consequences for everyday life. This may be because first cases are reported relatively early in the epidemic when many of these more serious mitigation strategies are not yet part of public discourse. Finally, although one might expect official news to reduce concerns related to false messages, we do not find much indication that state announcements of first COVID-19 cases affect searches questioning whether the epidemic may be a hoax. Overall, our analysis of internet search data suggests that government information disclosure does help focus public attention on the crisis. Our evidence also indicates that people seem to mainly react by seeking information on what they can and should do in response to the epidemic. One conjecture is that policy makers may be able to take advantage of a brief period of active information seeking following focal events (first cases and first deaths) to provide clear advice regarding the actions people can undertake to avoid risk.

## Materials and Methods

### Samples.

Our analysis is based on a balanced daily panel of COVID-19−related search intensity data in 50 US states and Washington, DC between January 1, 2020 and March 18, 2020 (*n* = 51 regions × 78 d). We collected the data using a restricted-access Google Health Trends Application Program Interface (API) account (10).

### Measures.

The outcome variables measure the daily share of all Google queries in a state that correspond to a particular term during our study period. We multiply the shares by 10 million and round to the nearest integer to make the measure more interpretable. We collected data on the timing of the first COVID-19 case announcements from media reports in each state; see Dataset S1. All states reported their first case by March 17, 2020.

### Data Analysis.

We estimated Poisson models in an event study (11) framework to examine the effect of the first COVID-19 case announcements on searches. In the model we use, the expected number of searches per 10 million is

$$y_{i,t}=\;e^{\left(\beta_{0}+\;\;\underset{k=\left[-41,31\right]}{\sum }\beta_{k}\;{\text{Announcement}}_{i,k}\;+\;\;\theta_{i}+\;\theta_{t}+\;\varepsilon_{it}\right)},$$

where ${\text{Announcement}}_{i,k}$ is an indicator variable set to 1 if the first case of state *i* was announced *k* days ago. The reference category was 1 d prior to the first case announcement, and we created single indicators ${\text{Announcement}}_{i,31}$, for any k≥30, and ${\text{Announcement}}_{i,-41}$, for any k≤−40. We controlled for state fixed effects to allow for time-invariant differences in search patterns by state, and for date fixed effects to account for national trends. SEs allowed for heteroskedasticity and clustering at the state level. Poisson coefficients and CIs are shown for the 40 d leading up to the first case announcement, and up to 20 d following it. The vertical axis measures the differential percentage change in search frequency as states approach the first case announcement (Figs. 1 and 2). We performed regression analysis using Stata, version 16.0 (StataCorp).

### Data and Code Availability.

State news data and code are available at https://github.com/anabento/GoogleBehaviorCovid (9). The search data for this study are available from Google Trends but are restricted in use; researchers may apply to Google Trends API for access.

## Limitations

Although the eventual failure of Google Flu Trends suggests that building and maintaining a complex model for a long period of time is difficult (12, 13), internet search volume has been shown to be a good proxy for many socioeconomic behavioral indicators, such as automobile sales (14) or dietary patterns (9). This study has, indeed, several limitations. First, our state-level data from Google Trends database do not allow us to adequately understand intrastate variation of search volumes following the announcements or to generalize conclusions about population search behavior (as Google Trends only captures the search behavior of a particular segment of information seekers). Second, the major issues with search query data—long-term drift and representation bias across geographic regions—does not come into play in our study because we focus on responses in a short time window surrounding the government announcements. The third limitation comes from potential confounding factors in our event study framework, including anticipatory behavior and unobservable local events (15). We mitigate these concerns by controlling for the timing of the announcements of Washington, Illinois, California, and Arizona as well as for the timing of the earliest announcement among a state’s neighbor states, in several sensitivity analyses.

## Supplementary Material

Supplementary File
